# Treatment Management Challenges in Naïve and Experienced HIV-1-Infected Individuals Carrying the M184V Mutation

**DOI:** 10.3390/v16091392

**Published:** 2024-08-30

**Authors:** Iordanis Mimtsoudis, Olga Tsachouridou, Karolina Akinosoglou, Symeon Metallidis

**Affiliations:** 1 Infectious Diseases Division 1st Internal Medicine Department, AHEPA University Hospital Thessaloniki, 54636 Thessaloniki, Greece; jordymim@hotmail.com (I.M.); metallidissimeon@yahoo.gr (S.M.); 2 Department of Internal Medicine and Infectious Diseases, University General Hospital of Patras, 26504 Patras, Greece; akin@upatras.gr

**Keywords:** HIV-1, drug resistance, M184V, mutation, naïve, experienced

## Abstract

M184V is a single-base mutation in the YMDD domain of reverse transcriptase (RT). The M184V resistance-associated mutation (RAM) is related to virological unresponsiveness to lamivudine (3TC) and emtricitabine (FTC) and induces high-level resistance to these two antiretroviral agents. M184V is rapidly selected in the setting of non-suppressive antiretroviral therapy (ART) and accumulates in the HIV reservoir. There were continuous efforts to evaluate the impact of the M184V mutation on the treatment outcomes in people living with HIV (PLWH). Since 3TC remains an extensively used part of recommended antiretroviral combinations, M184V is commonly detected in patients with virological failure (VF). ART guidelines do not recommend the use of drugs impacted by RAMs as they have been confirmed to comprise a risk factor for VF. However, there is evidence that 3TC/FTC can remain active even in the presence of M184V. Given the potential benefits of 3TC in ART combinations, the investigation of M184V remains of high interest to clinicians and researchers, especially in certain regions with limited resources, and especially for its unusual effects. This is a review of the literature on the challenges in treating both naïve and experienced individuals carrying the M184V mutation, including virological failure, virological suppression, and resistance to ART.

## 1. Introduction

The HIV epidemic remains a major public health concern and treating people living with HIV (PLWH) is accompanied by significant challenges despite current efficacious antiretroviral therapy (ART) [1]. The emergence of drug resistance due to the selection and/or transmission of HIV-1 variants harboring resistance-associated mutations (RAMs) is strongly correlated to high rates of virological failure (VF) in either ART-naïve or ART-experienced patients, which is defined as HIV viral load (VL) > 50 copies/mL at 6 months after starting therapy [2,3]. This phenomenon compromises the success of commonly used potent antiretroviral drugs. One of the most frequently encountered RAMs is the M184V mutation in the reverse transcriptase (RT) region of the *pol* gene which confers high-level resistance to lamivudine (3TC) and emtricitabine (FTC) and potentially leads to VF in PLWH being treated with regimens containing these antiretroviral agents, resulting in a limited number of available treatment options [3,4,5,6,7,8,9]. Therefore, genotypic resistance testing is extremely important prior to the initiation or modification of ART to obtain a viral resistance profile that can be used to guide appropriate treatment decisions [10]. In the case of demonstrated RAMs, current ART guidelines recommend against the use of drugs with documented resistance while choosing a regimen containing at least two, preferably three, fully active drugs. Nevertheless, several experts suggest that the continuation of treatment with 3TC or FTC might be beneficial despite the presence of the M184V mutation [10].

In this review article, we aim to summarize the history of 3TC/FTC and the identification of M184V, its impact on viral fitness, the current regional and treatment status epidemiology of M184V, and the implications in daily clinical practice. We conducted a systematic literature search in PubMed through March 2024, using keywords related to HIV, RAMs related to NRTIs, the M184V mutation, and outcomes in both treatment-naïve and -experienced individuals. Identified articles were manually searched for relevance. The prevalence of pretreatment or acquired mutations was reported separately. Pretreatment mutations refer to mutations that occurred in studies that described the individuals as treatment naïve. Acquired mutations refer to those that occur after VF.

## 2. Evolution of Antiretroviral Therapy

ART stands as one of modern medicine’s greatest achievements in combating the HIV epidemic. Over the past few decades, the landscape of ART has evolved dramatically, resulting in a significant alteration of the natural course of the HIV-1 infection, which is currently considered a manageable chronic condition [11].

The first breakthrough in ART came in the late 1980s with the approval of zidovudine (AZT), a nucleoside reverse transcriptase inhibitor (NRTI). AZT was a pioneer in HIV treatment, significantly delaying disease progression and reducing mortality rates. However, its efficacy was limited, and the virus quickly developed resistance when used as monotherapy [12].

The subsequent years witnessed a rapid expansion of the antiretroviral armamentarium, with the development of new drug classes and combination therapies. Protease inhibitors (PIs), another class of antiretroviral drugs, emerged in the mid-1990s and revolutionized HIV treatment by targeting the viral protease enzyme, essential for viral replication. Combination regimens, commonly referred to as highly active antiretroviral therapy (HAART), became the standard of care, consisting of two NRTIs combined with either a PI or a non-nucleoside reverse transcriptase inhibitor (NNRTI) [10,12].

The introduction of HAART marked a pivotal moment in the HIV epidemic, leading to dramatic reductions in HIV-related morbidity and mortality rates, allowing individuals to achieve viral suppression and restore immune function [10,12].

Despite the success of HAART, challenges persisted, including drug toxicity, pill burden, and the emergence of drug-resistant viral strains. As a response, researchers continued to innovate, developing new classes of antiretroviral drugs with improved efficacy, safety, and tolerability profiles [12].

The advent of integrase strand transfer inhibitors (INSTIs) in the late 2000s represented a significant milestone in HIV treatment. INSTIs, such as raltegravir (RAL), dolutegravir (DTG), and bictegravir (BIC), target the integration of viral DNA into the host cell genome, effectively blocking viral replication at a crucial stage of the viral life cycle. These agents demonstrated potent antiviral activity, a high barrier to resistance, and favorable side effect profiles, further enhancing treatment options for HIV-infected individuals [2,12].

Moreover, advances in drug delivery formulations, such as single-tablet regimens (STRs), have simplified treatment regimens, improving adherence and treatment outcomes. STRs combine multiple antiretroviral agents into a single pill, reducing pill burden and dosing frequency, which has been shown to enhance adherence and virological suppression rates [12,13].

Currently, the field of HIV therapeutics continues to evolve, with ongoing research focusing on novel treatment modalities, including long-acting injectable formulations, broadly neutralizing antibodies, and therapeutic vaccines; innovations that hold the promise of further transforming HIV treatment, offering new avenues for achieving sustained viral suppression and improving quality of life [2,12].

Despite these groundbreaking advancements, several challenges remain in managing HIV infection, particularly in populations with limited access to healthcare resources and those impacted by high rates of treatment failure or drug resistance [5,7,14]. Addressing these challenges by developing novel therapies, optimizing treatment strategies, and improving access to care for all PLWH is central to ongoing research efforts.

## 3. History of 3TC/FTC and Identification of M184V

3TC, a first-generation nucleoside reverse transcriptase inhibitor (NRTI) that was initially approved for the treatment of HIV-1 infection in 1995 and in 1998 for hepatitis B virus (HBV) infection [15]. It has been evaluated in more than 50 clinical studies involving up to 25,000 PLWH. Since 2002, both 3TC and FTC have been recommended components of most fixed-dose combinations by the WHO [16]. Moreover, guidelines continue to recommend 3TC as part of fixed-dose combinations as first- and second-line ART for PLWH of all ages [16]. In addition, in coinfection with HIV and HBV, the use of TDF/3TC or FTC is suggested for first-line ART regimens [17,18,19,20,21]. ART has evolved to consist of single-tablet regimens including three- or four-drug combinations, and 3TC remains a well-established component in main HIV treatment strategies as the infection evolves.

3TC wields its antiviral effect by acting like a DNA-chain terminator. It comprises a potent inhibitor of the reverse transcriptase (RT) enzymes of HIV, with limited toxicity to the cells at concentrations >1000-fold compared to those effective against HIV [22,23,24,25,26]. The unique 3TC chemical structure strongly contributes to its clinical success and presents excellent antiviral activity.

The primary identification of 3TC revealed that resistance to the agent develops rapidly in vitro [27]. Resistance to 3TC or FTC occurs via mutations compared with other NRTIs [28]. Mutations in codon 184 lead to the substitution of valine or isoleucine in place of the wild-type methionine through a nonsynonymous mutation (M184I or M184V) [27]. Amino acid 184 is located in the highly conserved tyrosine/methionine/aspartate/aspartate (YMDD) motif of HIV-1 reverse transcriptase and confers high-level resistance to 3TC, approximately more than 100-fold, which is necessary for the proper catalytic action of the enzyme [27,29]. Furthermore, the mutation was confirmed to increase susceptibility to zidovudine (ZDV) [30] and, subsequently, stavudine (d4T) [31] and to tenofovir [32]. In addition, M184V enhances fidelity and diminishes the handleability of HIV RT, leading to modest viral fitness by having an impact on the catalytic activity of RT. 

Wild-type RT exhibits 2- to 6-fold-increased mutation frequency compared to purified M184V variant proteins [33,34], probably accounting for the reduced rate of drug resistance. All these functions result in residual antiviral activity, even with 3TC monotherapy in patients who carry the M184V mutation [35]. In a prospective randomized 48-week study with 58 participants with virologic failure on a regimen containing 3TC and with confirmed treatment-emergent M184V interchange, clinical or immunological failure was confirmed in almost 70% of participants who discontinued ART and 41% of patients who continued monotherapy with 3TC (*p* = 0.064) [35]. Added to that, ten years earlier, in another study with nearly 2000 patients randomized to receive either 3TC, 3TC plus loviride, or a placebo added to their actual ART of either ZDV or ZDV and ddI, 3TC dropped the risk of disease progression by more than 50% compared to the placebo (HR, 0.42; *p* < 0.0001) [36]. Guidelines recommend considering the continuation of 3TC or FTC in certain cases even if M184V has been documented [16,37].

Clinical data from 132 treatment-experienced, virally suppressed individuals carrying multiple mutations (M184V included) in a more recent study demonstrated that boosted darunavir (DRV/r) or ritonavir-boosted lopinavir (LPV/r) plus 3TC was superior to boosted protease inhibitor (PI) monotherapy, with only 3% of patients facing virological failure after 48 weeks on treatment [38]. These outcomes speculate the hypothesis that the selection of M184V by 3TC leads to residual antiviral activity that, when combined with other antiretrovirals, could result in controlling viral replication.

FTC shares similar characteristics and safety profiles, as shown in head-to-head studies with 3TC [39,40,41]. By contrast, 3TC achieves higher intracellular levels of the active triphosphate analog than FTC [42]. Like 3TC, FTC alone has been confirmed to select for M184V in vitro, usually within 2 weeks upon initiation [43]. In a pooled analysis of trials in which the treatment-naïve patients received EFV plus two NRTIs containing either 3TC or FTC, the frequency of individuals with observed M184V variants was 1.0% in patients treated with FTC and nearly triple in patients receiving 3TC (*p* = 0.009) [44]. However, the conclusions cannot be generalized due to the cross-trial comparison nature of the study, consisting of different NRTI partners.

Both agents are frequently considered clinically equivalent [45]. However, in combined ART, 3TC- and FTC-containing combinations have comparable efficacy [46]. A recent review study assessed clinical trials that compared FTC and 3TC as part of combined regimens for treatment-naïve or experienced PLWH between 2002 and 2013 [46]. Twelve clinical trials provided 15 distinct randomized comparisons, providing data on more than 2000 participants receiving 3TC and more than 2500 patients receiving FTC. In those trials, treatment outcomes did not differ significantly. In three trials that compared FTC and 3TC directly, the RR of treatment success was non-significant (RR, 1.03; 95% CI: 0.96 to 1.10; P = 0.3). For all 12 trials, the pooled RR treatment success was not significantly different (RR, 1.00; 95% RR of treatment failure (RR, 1.08; 95% CI: 0.94 to 1.22)).

By contrast, a study of 4740 naïve HIV patients without baseline resistance from the ATHENA cohort run in the Netherlands, being treated with 3TC or FTC plus an NNRTI (EFV or nevirapine) and tenofovir, found that virologic failure occurred more often in patients treated with 3TC than in patients treated with FTC [odds ratio (OR; 95% CI) with nevirapine/tenofovir, 2.09 (1.25–3.52), *p* = 0.005; with EFV/tenofovir, 1.78 (1.11 to 2.84), *p* = 0.016] [47]. However, in another study from the ATHENA cohort of 1582 treatment-naïve patients with HIV who initiated treatment with 3TC or FTC with a boosted PI and TDF, no significant differences in the rates of virologic failure were observed in individuals administered 3TC compared with patients on FTC [38]. Overall, these findings support the current guidelines that consider FTC and 3TC as commutable for initial treatment; however, more prospective studies may be more efficacious in evaluating the interchangeable nature of FTC and 3TC on contemporary ART combinations.

## 4. Resistance and Impact of M184V on Viral Fitness

The efficacy of 3TC monotherapy was restricted by the primary development of a mutation causing the M184I substitution in reverse transcriptase and later with the emergence of M184V, detected within the first month of 3TC monotherapy in both treatment-naïve and -experienced patients [48,49]. Although HIV-1 RNA reduction occurred in the first 2–4 weeks of 3TC monotherapy, the selection of the M184V virus resulted in a rebound in viral load levels, leading to an overall viremia reduction of 0.5 logs after 48 weeks of initiation [48]. In vitro analysis of a heterogeneous selection of M184V and wild-type HIV strains showed that the relative concentration of M184V had to be at least 20% to result in a >1-fold increase in 3TC resistance and more than 50% to result in a fold change greater than 3TC’s clinical cutoff of 3.5 [50]. Interestingly, after the selection of M184V, some in vitro activity of 3TC was maintained when combined with other agents, even in the presence of full resistance to 3TC [51]. In vitro studies showed that the M184V variant incorporates deoxycytidine triphosphate instead of lamivudine 5′-triphosphate 20–100-fold more frequently than the wild-type virus [52]. This mechanism of resistance is in contrast with the mechanism of zidovudine resistance. Reverse transcriptase carrying ZDV resistance mutations still incorporates ZDV, but ZDV resistance mutations accelerate the rate of excision reaction [53]. The M184V mutation inverts the effect of ZDV resistance mutations by reducing the pace of ZDV excision [53]. Hence, the observed maintained response to 3TC/ZDV combined ART may be explained up to a point by the sensitization to ZDV by the M184V mutation [54]. The synergy between 3TC and ZDV played a crucial role in early suppressive therapies and set the beginning of the combined ART era with 3TC/ZDV, which comprised the first fixed-dose combination.

The M184V substitution in HIV-1 RT develops rapidly following the initiation of therapy with 3TC and confers high-level phenotypic resistance to this drug both in vitro and in vivo. Interestingly, the presence of M184V is also associated with the alteration of several mechanisms relating to RT function that include decreased RT processivity, reduced nucleotide-dependent primer unblocking, increased fidelity, hypersensitization to other NRTIs, impaired viral fitness, and the delayed appearance of mutations in RT that are responsible for resistance to thymidine analogs. In addition, M184V may affect viral transmission and immunological response. Collectively, these factors might explain the residual antiviral effect and clinical benefit observed with the continued use of 3TC in combination therapy regimens following the emergence of M184V.

Viral fitness is defined as the ability of a virus to adapt to its environment in terms of replicative capacity. Viruses with higher fitness can outcompete those of lower fitness, as measured by tissue culture assays including viral growth kinetics, single-cycle infection, and growth competition [55]. The diminished fitness of viruses containing the M184V mutation has been shown both in vitro [56,57] and in clinical studies [58,59,60,61].

Some studies have also demonstrated that a ~10% reduction in HIV replication activity leads to a reduction in viral fitness associated with the M184V variant. The presence of the M184V substitution further diminishes the viral fitness of HIV strains carrying resistance mutations confirmed to impact viral fitness [62,63]. The sedate replication of mutant viral strains may further diminish viral fitness in CD4+ lymphocytes [64].

Laboratory and preclinical data imply that the preservation of M184V by the sustained selection pressure of 3TC may be beneficial on clinical grounds by intercepting the overgrowth of wild-type virus, especially in cells with low deoxynucleotide triphosphate levels (like macrophages). It has been proposed that the lower replication activity of M184V variants could result in a restriction of HIV transmission. Although M184V is commonly acquired during treatment with NRTIs, it is rarely detected in treatment-naïve patients, suggesting that M184V variants are rarely transmitted [65]. According to a study that used population genetic sequencing, 67.5% of patients with virologic failure in first-line treatment harbored M184V variants compared with only 7% of those who were treatment-naïve [65].

The literature speculates that it is the diminished fitness that causes the elimination of M184V in the absence of selection by 3TC or FTC and that M184V does not substantially affect HIV transmission. Studies of NRTI regimens suggest a residual clinical benefit of 3TC despite the presence of M184V mutants [66,67]. For example, in NUCA 3001, plasma HIV-1 RNA levels remained >0.5 log10 below baseline in participants who received 3TC monotherapy despite the emergence of the M184V substitution and high-level phenotypic resistance [66]. Moreover, in a small pilot study, the withdrawal of 3TC in viremic patients with multidrug-resistant HIV and the M184V substitution led to a 0.5 log10 increase in viral load at 6 weeks, suggesting a persistent antiviral effect of 3TC despite the presence of the M184V substitution [68]. These and other clinical observations suggest a benefit of continuing 3TC even in the presence of M184V mutants; however, determining the clinical relevance of this benefit may require further study [67,69].

## 5. Global Epidemiology of M184V Mutation

The epidemiology of the M184V mutation is directly related to the dynamics of HIV-1 drug resistance, primarily affecting individuals with a history of ART exposure. Drug resistance often emerges in ART-experienced individuals due to selection pressure exerted by administered antiretroviral agents, enhancing the replication of mutated HIV-1 variants. This leads to increased viral load and eventual VF, contributing to acquired drug resistance. Subsequently, these mutations can be transmitted to newly infected individuals who have not yet received ART, resulting in transmitted drug resistance [2].

The prevalence of M184V is rather high in both low-/middle-income (LMICs) and high-income countries, especially acquired in ART-experienced individuals. Although it is not as common in ART-naïve compared to experienced patients, M184V/I is still prevalent in HIV individuals starting ART [70,71]. In naïve individuals, M184V/I has previously been detected in 1.2% (25/2436) of HIV patients in LMICs and among 54% and 31% of patients starting ART with NRTI resistance in LMICs and in high-income countries, respectively [72,73]. In a study, the prevalence of pre-treatment drug resistance was investigated among HIV-infected individuals initiating or re-initiating antiretrovirals in 63 countries. In this study, M184V/I accounted for the majority of NRTI resistance mutations, and the proportion doubled among those who restarted ART [74]. In treatment-experienced individuals, M184V/I accounts for the highest proportion of NRTI resistance worldwide [71]. M184V/I has been recorded in large proportions (53–92%) in LMICs [75,76] and in more variation (25–87%) among HIV adults in upper-income and high-income countries [4]. In individuals carrying TDF resistance, M184V/I has been observed in at least 83% of them [77].

According to a systematic review that investigated the prevalence of pretreatment and acquired HIV-1 mutations associated with drug resistance, the estimated global prevalence of acquired RAMs was 58% for any NRTIs, mainly at position M184, whereas the prevalence of pretreatment NRTI-associated mutations was significantly lower (4%), with M184V/I being among the most frequent (1%) [3]. Several challenges concerning the achievement and maintenance of viral suppression (adherence, drug resistance, stigma, depression, late diagnosis, discrimination, poor access to health care, and language barriers) remain and are linked to regional distribution [78,79,80,81,82]. These factors may lead to the development of DRMs, which may also subsequently lead to the transmission of drug-resistant strains [83].

There have been many reports that have included data on pretreatment and acquired DRM rates from different geographic locations and varied populations. Thus, there are many reports that can be analyzed to provide a comprehensive review of global and regional prevalence rates for DRMs.

Influenced by factors such as treatment availability, adherence patterns, and the local prevalence of drug-resistant strains, the prevalence of the M184V mutation varies geographically, as was previously shown by studies conducted in diverse regions, highlighting the significant prevalence of NRTI-associated mutations, with M184V emerging as a key mutation.

The estimated global prevalence of pretreatment DRMs was 4% (*n* = 2719/60,567; range, 2% (Asia Pacific) to 6% (North America)) for those associated with NRTI resistance [82,83,84,85,86,87,88,89,90,91,92,93,94,95,96,97,98,99,100,101,102]. The only pretreatment DRMs estimated to occur in >1% of the global population were 3TC/FTC-associated DRMs at position M184 (1%). Mutations at position M184 were the most frequent DRMs in Asia Pacific (1.6%) and the Middle East and North Africa (1.1%). The global estimated prevalence of acquired DRMs was 58% (*n* = 17,073/29,218) for resistance to any NRTI [87,103,104,105,106,107,108,109,110,111,112,113,114,115,116,117,118,119,120,121,122,123,124,125,126,127]. The highest rates of acquired DRMs for NRTIs were reported in Africa (NRTIs, 77%, *n* = 702/917) and the lowest were reported in North America (NRTIs, 23%, *n* = 86/378). In the subset of studies that enrolled patients only in the past 5 years (2013–currently), global estimates of acquired DRMs were consistent with the full data. Acquired NRTI DRMs were observed in 59% (*n* = 281/475) of participants. Across all regions, the most frequently acquired DRMs were the 3TC/FTC-associated mutations at position M184 (20%-Asia Pacific to 70% in Africa).

In studies from Europe, there were nearly 33,400 individuals with data associated with pre-treatment NRTI drug resistance mutations (DRMs) [57,68,70,90,97,128,129,130,131,132,133,134,135,136,137,138,139,140,141]. Pre-treatment DRMs with NRTI resistance were observed in 4% of the studied population. The most frequently observed pre-treatment mutation was M184 (1%). Regarding acquired mutations for resistance to NRTIs, resistance was observed in 70% of individuals (*n* = 1846/2633). M184 was the most frequently acquired 3TC/FTC DRM in Europe (51%; *n* = 1344/2633). A French study found that the most detected NRTI RAMs were M184V/I, with an estimated prevalence of 25.9% [4]. In Cameroon, NRTI-associated mutations were detected in 30% of ART-experienced individuals and 2.4% of ART-naïve individuals, with M184V being the most common mutation observed [5]. Similar findings were reported in a meta-analysis conducted in China, demonstrating high rates of M184V in both ART-treated and ART-naïve individuals [6]. Furthermore, studies in South Africa and India have underscored the widespread prevalence of NRTI-associated resistance mutations, with M184V being notably prevalent [7,8]. These mutations result in significant clinical management challenges and contribute to VF, complicating the treatment of PLWH and potentially facilitating the transmission of drug resistance [2,9].

Across regions, drug resistance mutation rates were broadly similar, with the highest rates of NRTI resistance in naïve individuals in North America (6%). Africa had the highest observed rates of acquired DRMs to any NRTIs (77%). Interestingly, a rather low rate of acquired NRTI mutations was recorded in North America (23%), compared with the respective global findings (58%). However, this difference should be interpreted with caution due to the limited number of individuals with available epidemiological data [142].

Comprehensively, pre-treatment data on the DRMs of the available population confirmed a mutation rate of 1% for MM184 is also among the most frequent positions for acquired drug mutations (51%). M184 mutations are known to confer resistance to 3TC/FTC but diminish viral fitness [143]. Subsequently, in an individual with a transmitted M184 virus, the DRM may not be detected if genotyping was performed after the acute phase, where the wild-type virus would outcompete M184 strains.

Surveillance efforts and molecular epidemiological studies are essential for monitoring trends in drug resistance mutations and designing public health strategies to mitigate their impact on HIV treatment outcomes.

## 6. Clinical Impact of M184V Mutation

The M184V mutation represents one of the most clinically significant mutations in the context of HIV-1 infection and ART [3,9]. It is located in the RT gene of the HIV-1 virus and induces high-level resistance to certain NRTIs, particularly 3TC and FTC, which indicates that the predicted level of resistance is similar to those observed in viruses with the highest levels of in vitro drug resistance and that clinical data exist demonstrating that patients infected with viruses having such mutations usually have little or no virological response to treatment with these drugs [144]. In contrast, M184V increases the susceptibility of the virus to tenofovir [9,145].

The clinical impact of the M184V mutation is multifaceted and has implications for both treatment-naïve and treatment-experienced individuals. Acknowledging its significance is extremely important for guiding treatment decisions and optimizing therapeutic outcomes.

In treatment-naïve patients, the presence of the M184V mutation can influence the selection of initial ART regimens [10]. Since M184V confers resistance to 3TC and FTC, which are commonly included in first-line regimens due to their efficacy and tolerability, the presence of this mutation may limit the choice of NRTIs [9]. This highlights the importance of baseline resistance testing to identify individuals with pre-existing resistance mutations, including M184V, and tailor treatment accordingly [8,10,14,145].

Furthermore, in treatment-experienced individuals, the emergence of the M184V mutation complicates the management of VF and the selection of salvage therapy. Reduced susceptibility to 3TC and FTC conferred by M184V may compromise the efficacy of these agents in subsequent treatment regimens. This necessitates the use of alternative NRTIs or other drug classes with preserved activity against M184V-resistant virus [10].

Moreover, the M184V mutation can impact the overall fitness of the virus and its replicative capacity. While M184V confers resistance to specific NRTIs, it may also result in reduced viral replication capacity and fitness, which could influence the trajectory of disease progression and the likelihood of treatment success. However, the clinical implications of these fitness costs require further investigation and may vary depending on the specific viral genetic background and host factors [2,9,145].

Additionally, the selection pressure exerted by NRTIs, particularly 3TC and FTC, may favor the emergence of additional resistance mutations, leading to multidrug resistance and further limiting treatment options [14,145].

## 7. Challenges in Naïve Patients

Detecting the M184V mutation in treatment-naïve individuals poses initial challenges due to cost constraints or local guidelines, potentially leading to suboptimal treatment outcomes. Limited treatment options arise from resistance to key antiretroviral components like 3TC and FTC, complicating regimen selection. Despite limited direct resistance to other drugs, such as tenofovir and abacavir (ABC), the presence of M184V can impede virological response to therapy, emphasizing the need for vigilant monitoring and early intervention in cases of VF [8].

The presence of M184V in ART-naïve patients not only restricts current treatment options but also raises the risk of cross-resistance to other antiretroviral agents [5,8]. The selection pressure from M184V and other mutations may prompt multidrug resistance, constraining future therapeutic choices. Therefore, the proactive detection of M184V and strategic management are crucial to prevent treatment failure and preserve future treatment avenues [8,145].

Recent research sheds light on the influence of the M184V mutation on VL during VF on first-line ART and its prevalence in patients experiencing VF on first-line NNRTI-based ART, particularly in Southern Africa.

One study delves into the effect of the M184V mutation on HIV-1 VL during the VF of first-line ART containing 3TC or FTC, tenofovir disoproxil fumarate (TDF), and an NNRTI. The study included 1445 participants from 32 study groups in 15 countries. Among these, 56.5% had the M184V mutation and exhibited higher median VL compared to those without the mutation. However, after adjusting for other mutations, M184V was no longer significantly linked to failure VL. The study also found that the compensatory mutation L74I, present exclusively in 10.2% of participants with M184V, may enhance viral replication, explaining higher VLs. Despite high-level resistance to 3TC and FTC and therefore reduced viral fitness, VLs were similar between 3TC-resistant and 3TC-susceptible viruses, indicating that compensatory mutations maintain replication efficiency. These findings underscore the complexity of ART resistance and suggest the need for clinical trials to assess the role of 3TC in second-line treatments for individuals with M184V mutations [145].

Complementing the previous study, a systematic review focusing on PLWH in Southern Africa experiencing VF on first-line NNRTI-based ART found high levels of the M184V/I mutation after two years, especially when combined with tenofovir or zidovudine. The prevalence of NRTI/NNRTI drug resistance mutations underscores the common occurrence of resistance in this population, influencing treatment decisions and the efficacy of second-line regimens. Despite NRTI resistance, DTG-based regimens may remain effective, as evidenced by studies where NRTI resistance did not hinder virological response to second-line regimens [14].

These findings highlight the intricate relationship between drug resistance mutations, viral replication capacity, and treatment outcomes in individuals failing first-line ART, particularly in regions with a high prevalence of drug resistance such as Southern Africa [14]. Understanding resistance patterns is crucial for refining treatment strategies and transitioning to DTG-based first-line ART in resource-limited settings [14].

The use of DTG-containing regimens in ART-naïve patients or as simplified therapy in patients carrying no resistance mutations to NRTIs, PIs, or INSTIs has been largely effective, with merely a few cases of resistance emergence in major DTG clinical trials. The LAMIDOL trial was established to evaluate the efficacy of DTG + 3TC in virologically suppressed adults on first-line triple combinations, in which no emerging resistance was recorded among participants [146]. The efficacy of DTG + 3TC in virologically suppressed patients was also evaluated in the ASPIRE study, with >90% of recruited patients (N = 89) achieving viral suppression and one patient experiencing virological failure at week 48 with no emerging resistance [147]. The simplified regimen of DTG + 3TC has also been evaluated in ART-naïve patients in the PADDLE, GEMINI1 and GEMINI 2, and ACTG A5353 studies. No VF or emerging resistance was registered in any of the study participants in either study (GEMINI1/GEMINI2 and PADDLE) [148,149]. The rare emergence of M184V mutation in ART-naïve patients initiated on DTG was reported in the ACTG A5353 pilot study, which evaluated DTG + 3TC efficacy in patients with pretreatment HIV-1 RNA up to 500,000 copies/mL [150]. The impact of M184V in treatment-naïve and virologically suppressed patients [151,152,153] carrying no resistance mutations is summarized in Table 1.

Therefore, routine resistance testing before initiating ART is highly recommended, particularly in settings with high HIV prevalence or known drug-resistant strains. This testing aids in identifying baseline mutations, including M184V, and guides regimen selection. However, challenges like access, cost, and infrastructure hinder widespread testing, necessitating innovative solutions and collaborative efforts among clinicians, researchers, and policymakers to ensure timely diagnosis, access to effective therapies, and comprehensive patient support [7].

## 8. Challenges in Experienced Patients

Treatment-experienced patients face unique challenges in managing HIV infection, particularly when harboring the M184V mutation. These individuals usually have been exposed to multiple antiretroviral drugs over time, thus increasing the likelihood of the accumulation of resistance mutations and further complicating the selection of salvage therapy.

Selecting an effective salvage therapy regimen in experienced patients with the M184V mutation requires careful consideration of the individual’s treatment history, resistance profile, comorbidities, and treatment preferences. Alternative NRTIs with activity against M184V-resistant virus, such as TDF or abacavir (ABC), may be considered [10].

In some cases, experienced patients with the M184V mutation may require a switch to alternative antiretroviral regimens due to virological failure, toxicity, or treatment intolerance. Switch strategies should consider the patient’s resistance profile, previous treatment history, and the availability of alternative agents with activity against the M184V-resistant virus. The close monitoring of treatment response and virological outcomes is essential following regimen switches to ensure adequate viral suppression and to prevent further development of drug resistance [10,13,154].

One significant challenge that arises when managing virally suppressed individuals prior to a regimen switch is the inability to perform RNA-based drug resistance testing due to a very low viral load, which does not allow for the adequate amplification of viral plasma RNA. This can be overcome by alternatively performing proviral DNA genotyping; a method using cellular HIV DNA derived from peripheral blood mononuclear cells (PBMCs) in order to obtain a comprehensive genotypic resistance profile [11].

A study investigating the behavior of drug-resistant viral variants (DRVs) harboring the M184V mutation in the proviral DNA of virally suppressed patients demonstrated that the persistence of the M184V mutation was associated with the duration and level of HIV-RNA replication under 3TC/FTC therapy. While the mutation decreased eventually over time in HIV-DNA, it was more frequently persistent in patients with a history of a longer duration of replication under 3TC/FTC. The study highlighted the importance of detecting DRVs for predicting VF and underscored the need for careful consideration when recycling drugs with viral activity potentially compromised by past resistance [155].

According to a recent publication, a consensus was reached among virologists and clinicians, who agreed, using the Delphi method, that a regimen containing a second-generation INSTI plus 3TC or FTC plus one NRTI confers a low risk of VF in a virally suppressed patient carrying the M184V substitution, documented on the current DNA genotype and/or on an RNA genotype performed within the last 5 years, for at least one year. However, when examining the combination of a second-generation INSTI plus 3TC or FTC, under the same conditions, virologists expressed a strong concern about significant VF risk over time, whereas clinicians did not reach a consensus [11].

Recent studies and a comprehensive review investigate the efficacy of switching to bictegravir/emtricitabine/tenofovir alafenamide (BIC/FTC/TAF) in maintaining virologic suppression among HIV-1 patients, including those with archived antiretroviral resistance, such as M184V/I mutations. Particularly, one study examined the efficacy of switching to BIC/FTC/TAF in maintaining HIV-1 RNA suppression among participants with archived antiretroviral resistance. Surprisingly high rates of pre-existing resistance substitutions, notably M184V/I mutations, were found among suppressed participants who switched to BIC/FTC/TAF. Despite this, high rates of virological suppression were maintained for up to 48 weeks with no development of resistance to study drugs. This study emphasized the importance of comprehensive resistance testing prior to treatment initiation or regimen switch, concluding that switching to BIC/FTC/TAF was noninferior to other regimens [154].

Another study specifically investigated the efficacy of switching to BIC/FTC/TAF in Black Americans with HIV-Results showed that only a few participants experienced HIV RNA levels of 50 copies/mL or higher through week 48 after switching to BIC/FTC/TAF, indicating a high rate of virologic suppression. Notably, participants with pre-existing resistance to NRTIs, including M184V/I mutation, achieved high rates of virologic suppression upon switching, with no development of treatment-emergent resistance during the study period. This study highlighted the real-world efficacy of BIC/FTC/TAF in maintaining virologic suppression, even in the presence of pre-existing NRTI resistance [156].

Furthermore, a review presented the recent literature supporting the use of BIC/FTC/TAF for consolidating therapy and maintaining virologic suppression, especially in individuals with M184V/I mutations based on randomized trials that demonstrated the efficacy of BIC/FTC/TAF in maintaining virologic suppression, even in participants with pre-existing M184V/I mutations. The review underscored the importance of medication adherence for sustaining virologic suppression on BIC/FTC/TAF [157].

These results are further supported by in vitro data, according to which viral strains carrying the M184V substitution exhibit diminished viral strength and heightened susceptibility to tenofovir. Thus, persisting to an emtricitabine/tenofovir-based therapy in cases involving the M184V mutation aims to uphold selective pressure, favoring a less robust virus that is particularly susceptible to tenofovir [9,154].

Moreover, another potential treatment option for experienced individuals harboring the M184V mutation is the abacavir/lamivudine/dolutegravir (ABC/3TC/DTG) regimen, as was demonstrated by a study that investigated the influence of the M184V/I mutation on virological outcomes in virally suppressed HIV-1 patients switching to ABC/3TC/DTG. The results indicated that patients with a documented M184V/I mutation tended to have a lower CD4 nadir and a longer history of antiviral therapy. Despite these differences, there was no significant association between the presence of M184V and the incidence of VF. These findings suggest that the ABC/3TC/DTG regimen effectively maintains virological suppression in treatment-experienced patients, regardless of M184V/I mutation status. The study highlights the potential of this regimen as a viable treatment option for individuals with a history of ART and documented M184V/I mutation. However, the authors emphasize the need for further research to validate these results over an extended follow-up period and explore any potential long-term impacts on treatment efficacy [158].

Additionally, one study explored the efficacy of switching to dolutegravir plus lamivudine (DTG + 3TC) in maintaining HIV viral suppression among aviremic individuals with a historical resistance to 3TC. At week 48, 92.7% of participants maintained virologic suppression with HIV-1 RNA levels of fewer than 50 copies/mL, and no cases of virologic failure were reported. Participants with historical 3TC resistance or the detection of 3TC resistance mutations achieved resuppression on DTG + 3TC, challenging the assumption that DTG + 3TC functions as DTG monotherapy in patients with a prior history of 3TC resistance. This suggests that 3TC may provide significant antiviral activity to DTG despite the presence of archived 3TC resistance mutations, underscoring the potential of DTG + 3TC as a reduced-drug regimen for the maintenance of HIV suppression in INSTI-naïve individuals with historical 3TC resistance, with careful assessment of baseline RAMs recommended [159].

Similarly, a meta-analysis based on five real-world studies and five interventional studies from Embase, Ovid Medline, Medline In-Process, and the Cochrane library investigated the impact of the M184V/I mutation on virologic response to DTG + 3TC in populations with suppressed HIV-Minimal impact of prior M184V/I mutations on virologic suppression was demonstrated after switching to DTG + 3TC, suggesting that 3TC-containing regimens, such as DTG + 3TC, can remain effective in individuals with historical M184V/I mutations, with low VF rates and no emergent INSTI resistance reported. This indicates that M184V/I mutations may have minimal impact on the effectiveness of DTG + 3TC as a switch strategy in virologically suppressed individuals with incomplete treatment histories, but further data are needed to explore the impact of various factors [160].

Previous reports show that patients with failure in first-line therapy or virologically suppression with M184V/I can reach or sustain virological suppression when switched to DTG + NRTIs combinations. Sörstedt E et al. showed that in most ART-experienced adults (220/374) with a history of resistance, the M184V/I mutation did not lead to failure on DTG + 3TC or to any drug resistance to DTG in those with VF [161]. In another study with 436 enrolments, switching to 3TC plus either boosted PI or INSTI, the previous detection of M184V did not lead more frequently to virological failure or treatment interruption [162]. Despite the presence of the M184V/I mutation in 63% of participants in the DODULAM trial, all of them remained virologically suppressed for 96 weeks of observation [163]. In a recent study of individuals, with pre-existing NRTI mutations (including M184V) in over half of them, virological suppression in nearly 97% was achieved with DTG + NRTI regimens after 1.5 years of follow-up, and no DRMs were traced [161]. In a retrospective observational study comparing the virological efficacy of boosted PI or INSTIs+ 3TC in patients with or without M184V, no VF was observed in *n* = 21 patients on 3TC + DTG followed up for a median of 10 months (IQR 6:14) [159]. Olearo et al. prospectively studied 1626 participants (137 harboring M184V/I) from Europe and only a low VF incidence rate difference (0.016, 95% CI; 0.014–0.05) between participants carrying or not carrying the M184V mutation [158].

In an observational, retrospective, multicenter study (DOLAMA) of 177 virologically suppressed patients switching to simplified DTG + 3TC for any reason, 96.7% had VL < 50 copies/mL using study protocol analysis and five had VF at week 48 [162]. Maintenance therapy of DTG + 3TC + ABC and cobicistat-boosted EVG + TDF + FTC in patients with M184V was investigated in a retrospective analysis of 75 individuals, 51 of whom had a previous genotype report. Despite 88% of patients in the genotype group carrying M184V, no VF was registered in the DTG group, but one highly ART-experienced patient with a history of poor adherence had VF in the EVG group after 2.3 years of follow-up [164].

In a retrospective study that assessed the efficacy of ABC/3TC/DTG in 154 virologically suppressed patients carrying the M184V/I mutation, no VF was registered in any of the study patients [165]. The DAWNING study *n* = 624 (312, DTG group, 315, LPV/r group) investigated the efficacy of DTG-based second-line ART in virologically failing patients (≥400 RNA copies/mL) and 90% carrying NRTI resistance. After 48 weeks of follow-up, 84% of patients in the DTG arm achieved viral suppression (HIV-RNA < 50 copies/mL) despite 71% of these patients harboring M184V [166].

In summary, these results provide evidence supporting the efficacy of DTG + 3TC in maintaining HIV viral suppression, even in individuals with historical resistance to 3TC, and suggest that the presence of M184V/I mutations may have minimal impact on the effectiveness of this regimen [159,160,167]. Outcomes from studies on treatment-experienced individuals carrying M184V are summarized in Table 2.

Despite significant advances in research regarding the efficacy of specific combinations of antiretroviral agents, equal emphasis should be placed on the adherence of individuals carrying the M184V mutation to ART to achieve and sustain virological suppression [5,10,13,157]. Regular monitoring of adherence, using methods like self-reporting and pill counts, could be a useful tool for the prevention of VF and the transmission of resistance due to poor patient adherence, while the monitoring of VL and CD4 cell counts aids in assessing treatment response, promptly detecting virological rebound, and guiding clinical decision-making [10,13].

## 9. Conclusions and Future Directions

The literature reviewed highlights the complexity of treatment management challenges faced by both naïve and experienced HIV-1 infected individuals carrying the M184V mutation [24]. For naïve patients, the emergence of transmitted drug resistance, particularly the prevalence of the M184V mutation, poses significant hurdles in achieving successful virological suppression with first-line ART [8,14,16,24]. Understanding the epidemiology of these mutations, especially in regions with high rates of drug resistance, is crucial for optimizing treatment strategies and transitioning to more effective regimens, such as DTG-based therapy, in resource-limited settings [7,14,167].

For experienced patients, recent studies have shown that the efficacy of switching to newer antiretroviral regimens, such as BIC/FTC/TAF, ABC/3TC/DTG, or DTG + 3TC, offers promising avenues for maintaining viral suppression despite historical RAMs, including M184V/I. These studies also underscore the importance of comprehensive resistance testing prior to treatment initiation or regimen switch to inform personalized therapeutic approaches. Moreover, ongoing research into the kinetics of archived M184V mutations in long-term virally suppressed patients provides answers regarding the persistence of RAMs and their impact on treatment outcomes over time [11,154,155,156,157,158,159,160,167].

Over the last years, as new antiviral agents and drug classes obtained approval, regimens that contain 3TC continue to be widely used as first-line treatment. In developing countries, 3TC is crucial in HIV care due to its great efficacy and safety profile and, nonetheless, the availability of low-cost generic products. Indeed, 3TC is recommended in almost all first line and a vast proportion of second-line combination regimens for all ages. Furthermore, 3TC has a well-formulated pharmacokinetic profile with minor interactions with other agents, which is also important even in the presence of multiple concomitant drugs in specific populations (i.e., the elderly). Among all drugs initially approved for HIV treatment, only 3TC continues to be included even in the latest global guidelines. With minimal drug interactions and low cost, 3TC continues to have a significant role in the latest strategies worldwide in combination with contemporary antiviral agents and remains an attractive component for future combination regimens.

M184V causes marked phenotypic resistance to 3TC/FTC, but this effect does not always translate clinically. HIV strains that harbor M184V are less fit. Virologic rebound occurs after the discontinuation of 3TC in patients who already had developed M184V; 3TC alone slows CD4 decline more than no treatment, despite the M184V mutation being present. M184V influences the in vitro susceptibility of certain other NRTIs in a favorable way (susceptibility to TDF, ZDV, and stavudine improves). In other words, an M184V-containing virus is more susceptible to tenofovir than a wild-type virus, a phenomenon referred to as hypersusceptibility. Due to these odd effects, and because both 3TC/FTC are well tolerated, there is a practice of continuing 3TC or FTC even in the presence of M184V.

This review tried to summarize all current knowledge on the laboratory and clinical impact of the M184V mutation. However, it has a few limitations. Only brief reports could be made for each perspective of interest, in order to be useful to the readers. Data on certain suggested treatment combinations are also not provided in this research. Illustrations are also limited due to the nature of the subject choice.

Looking ahead, future directions in HIV treatment management should prioritize strategies that address the expanding landscape of drug resistance, including the development of novel antiretroviral agents with improved resistance profiles. Additionally, efforts to enhance adherence to treatment regimens, monitor virological outcomes, and optimize therapeutic interventions tailored to individual patient needs are paramount. Collaborative efforts between clinicians, researchers, and public health officials are essential for advancing our understanding of the challenges posed by the M184V mutation and implementing evidence-based interventions to improve clinical outcomes and reduce the transmission of drug-resistant HIV strains [13].

## Figures and Tables

**Table 1:** DTG and NRTIs in ART naïve and treatment experienced patients.

Reference	Study Type	N of Study Participants	ART Regimen	Resistance Prior to Switch	VL Status during Switch	VL < 50 copies/m, 48 Week	VF/Emergence of Resistance
Cahn et al. (2017) [148]	PADDLE-pilot study	20	DTG + 3TC	No	Naive	18/20 (90%)	−/No
Maggiolo et al. (2017) [151]	Prospective cohort	94	DTG + 3TC	No	Suppressed	97%	−/No
Taiwo et al. (2018a) [147]	ASPIRE-open-label randomised multicenter	89	DTG + 3TC, DRVr + 3TC + ABC	No	Suppressed	90.9%-DTG + 3TC, 88.9%-DRVr + 3TC + ABC	One pt in DTG + 3TC/No
Taiwo et al. (2018b) [150]	ACTG A5353-phase 2, single arm, open-label	120	DTG + 3TC	No	Naive	3/120 (2.5%)	Three pts/Yes
Blanco et al. (2018) [152]	DOLAM-open label, non-inferiority, randomised controlled trial	91	DTG + 3TC, DTG mono, or control (triple ART)	No	Suppressed	100%-control, 96.6%-DTG + 3TC, 93.5%-DTG mono	2 in DTG monotherapy, 1 in DTG + 3TC/Yes
Joly et al. (2019) [146]	LAMIDOL-Open label single arm multicenter	104	DTG + 3TC	No	Suppressed	97% (95% CI: 94–100%) meeting design expectation	1/No
Cahn et al. (2019) [149]	GEMINI 1 and 2-double-blind, randomised, non-inferiority, phase 3	1433	DTG + 3TC, DTG + TDF + FTC	No	Naive	GEMINI 1 (90% vs. 93%) and GEMINI 2 (93% vs. 94%).	−/No
Van Wyk et al. (2020) [153]	TANGO-phase III, randomised, open-label active controlled, multicentre	741	DTG + 3TC, TAF based	No	Suppressed	93.2%-3TC + DTG, 93.0%-TAF based	−/No

ART, antiretroviral therapy; Mono, monotherapy; VF, virological failure; DTG, dolutegravir; FTC, emtricabine; 3TC, lamivudine; TDF, tenofovir; ABC, abacavir; TAF, tenofovir alafenamide; DRV/r, darunavir/ritonavir; NRTIs, nucleoside reverse transcriptase inhibitors.

**Table 2:** DTG and NRTIs in treatment experienced patients with history of M184V.

Reference	Study Type	N of Study Participants	ART Regimen	VL Status at Switch	Presence of M184V/I at Switch	Resistance Prior to Switch	Viral Load < 50 copies/mL	Emerging Resistance at 48 Weeks	Virological Outcome Affected by M184V
Borghetti et al. (2016) [168]	Retrospective observational	36	DTG + 3TC	Suppressed	3 (8.3%)	Yes	100% ^a^	N/A	N/A
Reynes et al. (2017) [163]	DODULAM-Pilot, monocenter, prospective	27	DTG + 3TC	Suppressed	10 (37%)	Yes	100% at week 96	N/A	N/A
Capetti et al. (2017) [169]	Observational multicenter	130	DTG + DRV/r	Suppressed/virological failure	81 (62.3%)	Yes	Rise to 90.8% from 60% at baseline	No	N/A
Gagliardini et al. (2018) [170]	Retrospective observational	436	3TC + PI/r, 3TC + INSTI	Suppressed	87 (20%)	Yes	91.9%-no M184 group, 87.8%-M184 group ^b^	N/A	N/A
Sörstedt et al. (2018) [161]	Retrospective	244	DTG + NRTIs, PI/r + NRTIs	Suppressed	95 (36.5%)-DTG group, 95 (27.7%)-PI/r group	Yes	96.7%-DTG group at week 78, and 97.5%-PI/r at week 75	No	N/A
Olearo et al. (2019) [158]	Observational longitudinal analysis	1626	DTG + 3TC + ABC	Suppressed	137 (8.4%)	Yes	N/A	No	No
Aboud et al. (2019) [166]	DAWNING-Phase 3b open-label, non-inferiority trial	627	DTG + NRTIs, LPV/r + NRTIs	Not suppressed	513 (82%)	Yes	261 (84%)-DTG group, 219 (70%)-LPV/r group at week 48	Yes ^d^	No
Borghetti et al. (2019) [171]	Observational single center	494	DTG + 3TC, ATV/DRV/r + 3TC	Suppressed	16 (8.7%)-DTG group, 39 (12.5%)-PI/r group	Yes	48.3%, 70.9%, 82.6% in DRV/r, ATV/r, and DTG respectively at week 96	N/A	No
Hidalgo-Tenorio et al. (2019) [162]	Observational, retrospective, multicenter (DOLAMA)	177	DTG + 3TC	Suppressed	4/90 (4.4%)	Yes	96.7% at week 48	Yes	No
Baldin et al. (2019) [172]	Retrospective	221	DTG + 3TC	Suppressed	20/187 (10.7%)	Yes	95.3%	No	N/A
Jary et al. (2020) [165]	Retrospective	154	ABC/3TC/DTG	Suppressed	154 (100%)	Yes	100% at 12 months	No	No
Galizzi et al. (2020) [173]	Single center, observational, retrospective	374	DTG + 3TC, DTG + RPV	Suppressed	60 (27%)	Yes	97.8%-DTG + 3TC, 95.1%-DTG + RPV ^c^	No	No
De Miguel et al. (2020) [159]	Open-label, single arm, 48-week pilot trial (ART-PRO)	41 with 21 having history of 3TC resistance	DTG + 3TC	Suppressed	15/21 at 5% NGS threshold, 20/21 at 1% NGS threshold	Yes	94.7%-history of 3TC resistance group, and 100%-no historical 3TC resistance	No	N/A

N/A, not applicable; NGS, next generation sequencing; ART, antiretroviral therapy; DTG, dolutegravir; 3TC, lamivudine; ABC, abacavir; RPV, rilpivirine; ATV, atazanavir; DRV, darunavir; LPV/r, lopinavir/ritonavir; DRV/r, darunavir/ritonavir; PI/r, protease inhibitor/ritonavir; NRTIs, nucleoside reverse transcriptase inhibitors. ^a^ At 127.3 person-months of follow up; ^b^ Free from virological failure; ^c^ Viral suppression estimated probabilities at week 48; ^d^ Emerging resistance at week 52.
